# Dynamics of Alliance Formation and the Egalitarian Revolution

**DOI:** 10.1371/journal.pone.0003293

**Published:** 2008-10-01

**Authors:** Sergey Gavrilets, Edgar A. Duenez-Guzman, Michael D. Vose

**Affiliations:** 1 Department of Ecology and Evolutionary Biology, University of Tennessee, Knoxville, Tennessee, United States of America; 2 Department of Mathematics, University of Tennessee, Knoxville, Tennessee, United States of America; 3 Department of Electrical Engineering and Computer Science, University of Tennessee, Knoxville, Tennessee, United States of America; 4 National Institute for Mathematical and Biological Synthesis, University of Tennessee, Knoxville, Tennessee, United States of America; Lund University, Sweden

## Abstract

**Background:**

Arguably the most influential force in human history is the formation of social coalitions and alliances (i.e., long-lasting coalitions) and their impact on individual power. Understanding the dynamics of alliance formation and its consequences for biological, social, and cultural evolution is a formidable theoretical challenge. In most great ape species, coalitions occur at individual and group levels and among both kin and non-kin. Nonetheless, ape societies remain essentially hierarchical, and coalitions rarely weaken social inequality. In contrast, human hunter-gatherers show a remarkable tendency to egalitarianism, and human coalitions and alliances occur not only among individuals and groups, but also among groups of groups. These observations suggest that the evolutionary dynamics of human coalitions can only be understood in the context of social networks and cognitive evolution.

**Methodology/Principal Findings:**

Here, we develop a stochastic model describing the emergence of networks of allies resulting from within-group competition for status or mates between individuals utilizing dyadic information. The model shows that alliances often emerge in a phase transition-like fashion if the group size, awareness, aggressiveness, and persuasiveness of individuals are large and the decay rate of individual affinities is small. With cultural inheritance of social networks, a single leveling alliance including all group members can emerge in several generations.

**Conclusions/Significance:**

We propose a simple and flexible theoretical approach for studying the dynamics of alliance emergence applicable where game-theoretic methods are not practical. Our approach is both scalable and expandable. It is scalable in that it can be generalized to larger groups, or groups of groups. It is expandable in that it allows for inclusion of additional factors such as behavioral, genetic, social, and cultural features. Our results suggest that a rapid transition from a hierarchical society of great apes to an egalitarian society of hunter-gatherers (often referred to as “egalitarian revolution”) could indeed follow an increase in human cognitive abilities. The establishment of stable group-wide egalitarian alliances creates conditions promoting the origin of cultural norms favoring the group interests over those of individuals.

## Introduction

Coalitions and alliances (i.e., long-lasting coalitions) are often observed in a number of mammals including hyenas, wolves, lions, cheetahs, coatis, meerkats, and dolphins [1]. In primates, both kin and non-kin, and both within-group and group-level coalitions are a very powerful means of achieving increased reproductive success via increased dominance status and access to mates and other resources [1]–[7]. In humans, coalitions occurs at many different levels (ranging from within-family to between-nation states) and represent probably the most dominant factor in social interactions that has shaped human history [8]–[15].

The evolutionary forces emerging from coalitionary interactions may have been extremely important for the origin of our “uniquely unique” species [16], [17]. For example, it has been argued that the evolution of human brain size and intelligence during Pleistocene was largely driven by selective forces arising from intense competition between individuals for increased social and reproductive success (the “social brain” hypothesis, also known as the “Machiavellian intelligence” hypothesis; [16]–[27]). Coalition formation is one of the most powerful strategies in competitive interactions and thus it should have been an important ingredient of selective forces acting in early humans. Moreover, one can view language as a tool that originally emerged for simplifying the formation and improving the efficiency of coalitions and alliances. It has also been argued that the establishment of stable group-wide egalitarian alliances in early human groups should have created conditions promoting the origin of conscience, moralistic aggression, altruism, and other norms favoring the group interests over those of individuals [28]. Increasing within-group cohesion should also promote the group efficiency in between-group conflicts [29], [30] and intensify cultural group selection [31].

In spite of their importance for biological, social and cultural evolution, our understanding of how coalitions and alliances are formed, maintained and break down is limited. Existing theoretical approaches for studying coalitions in animals are deeply rooted in cooperative game theory, economics, and operations research [32]–[35]. These approaches are usually limited by consideration of coalitions of two individuals against one, focus on conditions under which certain coalitions are successful and/or profitable and assume (implicitly or explicitly) that individuals are able to evaluate these conditions and join freely coalitions that maximize their success [36]–[44]. As such, they typically do not capture the dynamic nature of coalitions and/or are not directly applicable to individuals lacking the abilities to enter into binding agreements and to obtain, process, and use complex information on costs, benefits, and consequences of different actions involving multiple parties [45]. These approaches do not account for the effects of friendship and the memory of past events and acts which all are important in coalition formation and maintenance. Other studies emphasize the importance of Prisoner's Dilemma as a paradigm for the emergence of cooperative behavior in groups engaged in the public goods game [46], [47]. These studies have been highly successful in identifying conditions that favor the evolution of cooperation among unrelated individuals in the face of incentives to cheat. Prisoner's Dilemma however is often not appropriate for studying coalitionary behavior [48], [49] especially when individuals cooperate to compete directly with other individuals or coalitions [16], [17] and within-coalition interactions are mutualistic rather than altruistic and the benefit of cooperation is immediate. The social network dynamics that result from coalition formation remain largely unexplored.

Here, we propose a simple and flexible theoretical approach for studying the dynamics of alliance emergence applicable where game-theoretic methods are not practical. Our method is related to recent models of social network formation and games on graphs with dynamic linking [50]–[55]. In our novel approach, alliances are defined in a natural way (via affinity matrices; see below) and emerge from low-level processes. The approach is both scalable and expandable. It is scalable in that it can be generalized to larger groups, or groups of groups, and potentially applied to modeling the origin and evolution of states [11]–[15], [56], [57]. It is expandable in that it allows for inclusion of additional factors such as behavioral, genetic, social, and cultural features. One particular application of our approach is an analysis of conditions under which intense competition for a limiting resource between individuals with intrinsically different fighting abilities could lead to the emergence of a single leveling alliance including all members of the group. This application is relevant with regard to recent discussions of “egalitarian revolution” (i.e. a rapid transition from a hierarchical society of great apes to an egalitarian society of human hunter-gatherers, [10]), and whether it could have been triggered by an increase in human cognitive abilities [16], [17].

## Methods

We consider a group of *N* individuals continuously engaged in competition for status and/or access to a limited resource. Individuals differ with regard to their fighting abilities *S_i_* (1<*i*<*n*). The attitude of individual *i* to individual *j* is described by a variable *x_ij_* which we call affinity. We allow for both positive and negative affinities. Individual affinities control the probabilities of getting coalitionary support (see below). The group state is characterized by an *N*×*N* matrix with elements *x_ij_* which we will call the affinity matrix.

Time is continuous. Below we say that an event occurs at rate *r* if the probability of this event during a short time interval *dt* is *rdt*.

We assume that each individual gets engaged in a conflict with another randomly chosen individual at rate α which we treat as a constant for simplicity. Each other member of the group is aware of the conflict with a constant probability *ω*. [Note that the value of *ω* can be affected both by the external environment and by biological and/or social characteristics of individuals.] Each individual, say individual *k*, aware of a conflict between individuals *i* and *j* (“initiators”), evaluates a randomly chosen initiator of a conflict, say, individual *i*, and helps him or not with probabilities *h_ki_* and 1−*h_ki_*, respectively. In the latter case, individual *k* then evaluates the other initiator of the conflict and helps him or not with probabilities *h_kj_* and 1−*h_kj_*, respectively. We note that the coalitionary support may be vocal rather than physical [58]. The interference probability *h_ij_* is given by an S-shaped function of affinity *x_ij_* and is scaled by two parameters: *β* and *η*. A baseline interference rate *β* controls the probability of interference on behalf of an individual the affinity towards whom is zero; *β* can be viewed as a measure of individual aggressiveness (i.e., the readiness to interfere in a conflict) or persuasiveness (i.e., the ability to attract help). A slope parameter *η* controls how rapidly the probability of interference increases with affinity. In numerical simulations we will use function
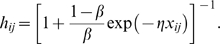



Notice that the probability of help *h_ij_* changes from zero to *β* to one as affinity *x_ij_* changes from large negative values to zero to large positive values. Below we will graphically illustrate the group state using matrices with elements *h_ij_* which we will call interference matrices.

For simplicity, we assume that interference decisions are not affected by who else is interfering and on which side. We also assume that individuals join coalitions without regard to their probability of winning. This assumption is sensible as a first step because predicting the outcomes of conflicts involving multiple participants and changing alliances would be very challenging for apes and hunter-gatherers.

As a result of interference, an initially dyadic conflict may transform into a conflict between two coalitions. [Here, coalition is a group of individuals on the same side of a particular conflict.] The fighting ability *S_I_* of a coalition *I* with *n* participants is defined as *s̅* *_n_n* ^2^, where *s̅* *_n_* is the average fighting ability of the participants. This formulation follows the classical Lanchester-Osipov square law [59]–[61] which captures a larger importance of the size of the coalition over the individual strengths of its participants. The probability that coalition *I* prevails over coalition *J* is set to *S_I_*/(*S_I_*+*S_J_*).

Following a conflict resolution we update the affinities of all parties involved by a process analogous to reinforcement learning [62]. The affinities of winners are changed by *δ_WW_*, of the losers by *δ_LL_*, the affinities of winners to losers by *δ_WL_*, and those of losers to winners by *δ_LW_*. The *δ*-values reflect the effects of the costs and benefits of interference on future actions. It is natural to assume that the affinities of winners increase (*δ_WW_*>0) and those of antagonists decrease (*δ_WL_*<0, *δ_LW_*<0). The change in the affinities of losers *δ_LL_* can be of either sign or zero. Parameters *δ_WW_*, *δ_WL_*, *δ_LW_*, *δ_LL_* are considered to be constant. We note that a negative impact of costs of interfering in a conflict on the probability of future interferences can be captured by additionally reducing the affinities of coalition members to its “initiator” by a fixed value *δ*.

We assume that coalitions are formed and conflicts are resolved on a time-scale much faster than that of conflict initiation. Finally, to reflect a reduced importance of past events relative to more recent events in controlling one's affinities, we assume that affinities decay towards zero at a constant rate *μ* [63]. Table 1 summarizes our notation.

**Table 1:** A summary of variables, parameters, functions, and statistics.

**Main dynamic variables**	
*x_ij_*	affinity of individual *i* to individual *j*
**Parameters**	
*N*	group size
*s_i_*	fighting ability of individual *i*
α	conflict initiation rate
ω	awareness
β	baseline interference rate
η	slope parameter
*δ_WW_*, *δ_WL_*, *δ_LW_*, *δ_LL_*	changes in affinity after conflict resolution
μ	affinity decay rate
κ	strength of social network inheritance
γ	birth rate
**Variables, functions, and statistics**	
*h_ij_*	probability that individual *i* helps individual *j*; is given by an S-shaped function of affinity *x_ij_* with parameters β and η
*S_I_* = *s̅* *n* ^2^	strength of coalition I with *n* members and average fighting ability *s̅*
*S_I_*/(*S_I_*+*S_J_*)	probability that coalition I wins a conflict with coalition J
*X_i_*	proportion of conflicts won by individual *i* since birth
*Y_i_* = Σ*b_k_*/*A_i_*	expected social success of individual *i*; *A_i_* is the age of individual *i* and *b_k_* is the benefit of the *k*th conflict
*H_X_*, *H_Y_*	standard deviations of *X_i_* and *Y_i_* in the group
*C* ^(1)^, *C* ^(2)^, *h̅*	clustering coefficients and the average probability of help in an alliance

## Results

To gain intuition about the model's behavior we ran numerical simulations with all affinities initially zero. We analyzed the structure of the interference matrix *h_ij_*, looking for emerging alliances. We say individuals *i* and *j* are allies if their interference probabilities *h_ij_* and *h_ji_* both exceed the baseline interference rate *β* by at least 50%. An alliance is a connected network of allies.

We also measured a number of statistics including the average and variance of affinities, the proportion of individuals who belong to an alliance, the number and sizes of alliances, the average interference probabilities *h̅* for all alliances present, and the clustering coefficients *C* ^(1)^ and *C* ^(2)^ [64] related to the probability that two allies of an individual are themselves allies. The average interference probability and the clustering coefficients can be interpreted as measuring the “strength” of alliances.

To make interpretation of model dynamics easier, we computed the proportion *X_i_* of conflicts won since birth, and the expected social success *Y_i_* = Σ*b_k_*/*A_i_*, where *A_i_* is the age of individual *i*, the sum is over all conflicts *k* he has participated in, the benefit *b_k_* is 1/*n_k_* if *i* was a member of a winning coalition of *n_k_* individuals, and *b_k_* is *0* if *i* was on the losing side. Although in our model the probability of winning always increases with the coalition size, the benefit *b_k_* always decreases with the coalition size. The net effect of the alliance size on the expected benefits of its members will depend on the sizes and composition of all alliances in the group. Note that our interpretation of *Y_i_* as a measure of expected social success makes sense both if all members on the winning side share equally the reward or if the spoils of each particular conflict goes to a randomly chosen member of the winning coalition. The former may be the case when the reward is an increase in status or rank. The latter may correspond to situations similar to those in baboons fighting over females, where members of the winning coalition may race to the female and whoever reaches her first becomes the undisputed consort for some time [48]. Non-equal sharing of benefits can be incorporated in the model in a straightforward way. Note also that being a member of a losing coalition always reduces relative social success.

We also calculated the standard deviations *H_X_* and *H_Y_* of *X_i_* and *Y_i_* values. These statistics measure the degree of “social inequality” in the group.

**Figure 1:**
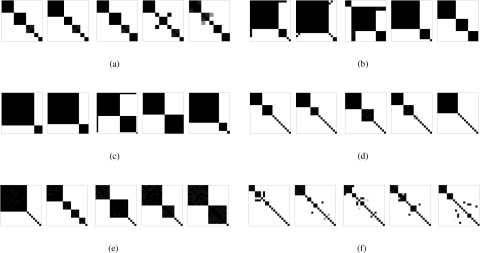
Interference matrices at time 1000. This figure shows the *NxN* interference matrices using small squares arranged in an *NxN* array with each of the small squares color-coding for the corresponding value of *h_ij_* using the gray scale from 0 (white) to 1 (black). The diagonal elements set to black for convenience so that the smallest squares on the diagonal represent unaffiliated individuals. For display purposes, alliances are ordered according to their clustering coefficients so that stronger alliances occur first along the diagonal. Parameters have default values except where noted. For each parameter combination shown are matrices observed in five different runs. (a) *N* = 10. (b) *N* = 20. (c) *N* = 30. (d) *N* = 20, *δ_LL_* = −0.5. (e) *N* = 20, μ = 0.1. (f) *N* = 20, ω = 0.25.

Figure 1 illustrates some coalitionary regimes observed in simulations using a default set of parameters (*α* = 1, *β* = 0.05, *δ_WW_* = 1, *δ_LL_* = 0.5, *δ_WL_* = −0.5, *δ_LW_* = −0.5, *η* = 0.5, *ω* = 0.5, *μ_a_* = 0.05) unless noted otherwise. This figure shows the *N*×*N* interference matrices using small squares arranged in an *N*×*N* array with each of the squares color-coding for the corresponding value of *h_ij_* using the gray scale. The squares on the diagonal are painted black for convenience. In all examples, individual strengths *s_i_* are chosen randomly and independently from a uniform distribution on [0,10] resulting in strong between-individual variation.

### Emergence of alliances

In our model, the affinity between any two individuals is reinforced if they are on a winning side of a conflict and is decreased if they are on the opposite sides; all affinities also decay to zero at a constant rate. The resulting state represents a balance between factors increasing and decreasing affinities. Although the emergence of alliances is in no way automatic, simulations show that under certain conditions they do emerge. The size, strength, and temporal stability of alliances depend on parameters and may vary dramatically from one run to another even with the same parameters. However, once one or more alliances with high values of *C* ^(1)^, *C* ^(2)^ and *h̅* are formed, they are typically stable. Individuals belonging to the same alliance have very similar social success which is only weakly correlated with their fighting abilities. That is, the social success is now defined not by the individual's fighting ability but by the size and strength of the alliance he belongs to. Individuals from different alliances can have vastly different social success, so that the formation of coalitions and alliances does not necessarily reduce social inequality in the group as a whole.

### Phase transition

**Figure 2:**
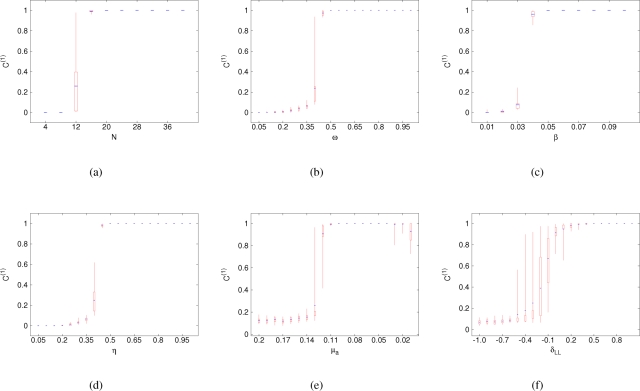
Tukey plots for the effects of *N*, *ω*, *β*, *η*, *δ_LL_* on the *C* ^(1)^ measure of the largest alliance. Each graph shows the effect of changing a single parameter from its default value (results for each parameter value are averaged over 20 runs, using data from time 1000 to 2000). The vertical lines extend from minimum to maximum observations, the dashed lines depict averages, and the boxes extend from lower to upper quartiles.

We performed a detailed numerical study of the effects of individual parameters of the properties of the system. As expected, increasing the frequency of interactions (which can be achieved by increasing the group size *N*, the awareness probability *ω*, baseline interference rate *β*, or the slope parameter *η*) and reducing the affinity decay rate *μ* all promote alliance formation. Most interestingly, some characteristics change in a phase transition-like pattern as some parameters undergo small changes. For example, Figure 2 show that increasing *N*, *ω*, *β*, *η*, or decreasing *μ* result in a sudden transition from no alliances to at least one very strong alliance with all members always supporting each other. Parameter *δ_LL_* has a similar but less extreme effect, whereas parameters *δ_WL_* and *δ_LW_* have relatively weak effects (Figures S1, S2, S3, S4, S5, S6, S7 and S8). Similar threshold-like behavior is exhibited by the *C* ^(2)^-measure, the average probability of help *h̅* within the largest alliance, the number of alliances, and the numbers of alliances with *C* ^(1)^>0.5 and with *h̅*>0.5 (see Figures S1, S2, S3, S4, S5, S6, S7, S8, S9 and S10). Interestingly, formation of multiple alliances is hindered when affinities between individuals fighting on the same side decrease as a result of losing (i.e., if *δ_LL_*<0).

### Cultural inheritance of social networks

Next, we extended the model to larger temporal scales by allowing for birth/death events, and the cultural inheritance of social networks. New individuals are born at a constant rate *γ*. Each birth causes the death of a different randomly chosen individual. We explored two rather different scenarios of cultural inheritance. In the first, the offspring inherits the social network of its parent who is chosen among all individuals with a probability proportional to the rate of social success *Y_i_*. This scenario requires special social bonds between parents and offspring. In the second, each new individual inherits affinities of its “role model” (chosen from the whole group either with a uniform probability or with a probability proportional to the rate of social success *Y_i_*). Under both scenarios, if individual *i* ^*^ is an offspring (biological in the first scenario or cultural in the second scenario) of individual *i*, then we set x_i__*j_ = *κx_ij_* for each other individual *j* in the group (parameter 0<*κ*<1 controls the strength of social network inheritance). In the parent-offspring case, the affinities of other individuals to the offspring are proportional to those to the parent: 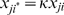 and 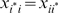 is set to *κ* times the maximum existing affinity in the group. In the role model case, other individuals initially have zero affinities to the new member of the group: 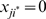.

### Stochastic equilibrium

**Figure 3:**
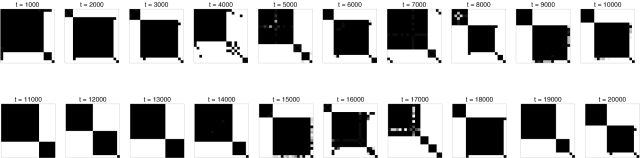
An example of the dynamics of an interference matrix in a stochastic equilibrium with no cultural inheritance (*κ* = 0). Parameters values are default with *N* = 20 and γ = 0.001 (so that the average life span is 1000). See the legend of Fig. 1.

If cultural inheritance of social networks is weak (κ is small), a small number of alliances are maintained across generations in stochastic equilibrium (see Figure 3). This happens because the death of individuals tends to decrease the size of existing alliances while new individuals are initially unaffiliated and may form new affinities. This stochastic regime is similar to coalitionary structures recently identified in a community of wild chimpanzees in Uganda [6] and in populations of bottlenose dolphins in coastal waters of Western Australia [65] and eastern Scotland [66].

### Egalitarian state

If cultural inheritance of social networks is faithful (κ is large), the dynamics become dramatically different due to intense selection between different alliances.

**Figure 4:**
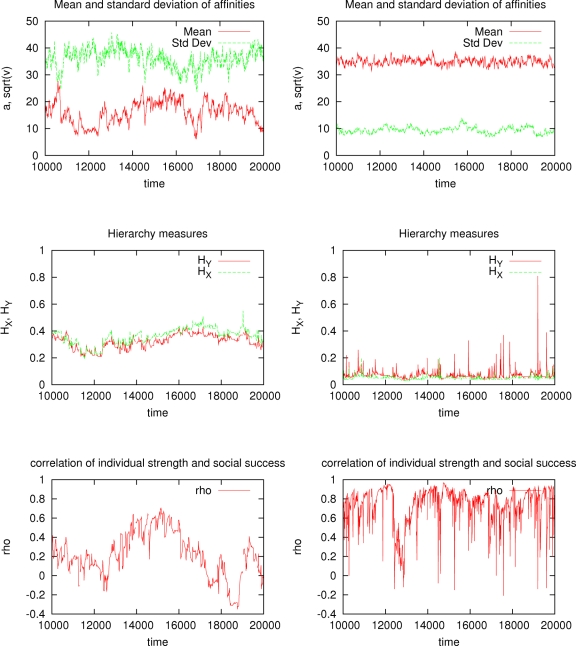
The graphs in the first column (Figures a, c and e) correspond to the run shown in Figure 3 (with *κ* = 0) which resulted in a small number of alliances maintained in stochastic equilibrium. The graphs in the second column (Figures b, d and f) correspond to a run with *κ* = 1 (complete cultural inheritance of social network) and *μ* = 0.025 (increased memory of past events) which resulted in an egalitarian regime. With several alliances present simultaneously (Figures a, c and e), the average affinity *a* is small, the variance of affinities *v* is large, the measures of social inequality *H_X_* and *H_Y_* are large, and the correlation between social success *Y_i_* and individual fighting ability *s_i_* is small. In the egalitarian state (Figures b, d and f), the average affinity *a* is large, the variance of affinities *v* is small, the measures of social inequality *H_X_* and *H_Y_* are small, and the correlation between social success *Y_i_* and individual fighting ability *s_i_* is large.

Now the turnover of individuals creates conditions for growth of alliances. Larger alliances increase in size as a result of their members winning more conflicts, achieving higher social success, and parenting (biologically or culturally) more offspring who themselves become members of the parental alliance. As a result of this positive feedback loop (analogous to that of positive frequency-dependent selection), the system exhibits a strong tendency towards approaching a state in which all members of the group belong to the same alliance and have very similar social success in spite of strong variation in their fighting abilities. Figure 4 contrasts an egalitarian state with the stochastic equilibrium illustrated in Figure 3 above. One can see that at the egalitarian state, the average affinity is increased while the standard deviation of affinity and the hierarchy measures are decreased. Although at the egalitatian state the correlation of individual strength and social success can be substantial, it does not result in social inequality. This “egalitarian” state can be reached in several generations.

### Cycling

**Figure 5:**
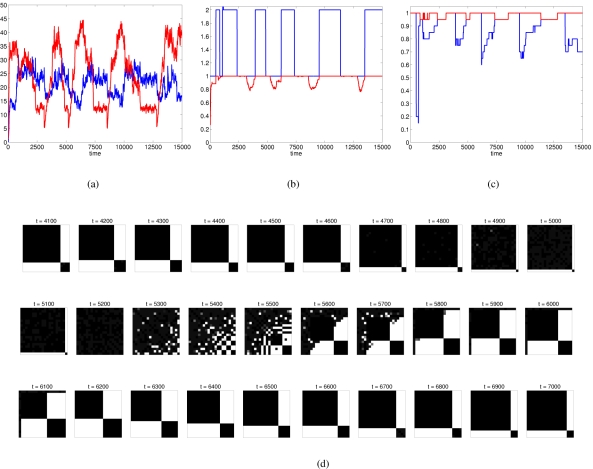
An example of a coalitionary cycle with complete cultural inheritance (κ = 1). Other parameters are as in Figure 3. (a) Average (blue) and standard deviation (red) of affinities in the group. (b) Number of alliances (blue) and clustering coefficient *C* ^(1)^ for the largest alliance (red). (c) Proportions of individuals belonging to an alliance (red) and to the largest alliance (blue). (d) Dynamics of the interference matrix between time 4100 and 7200.

However, the egalitarian state is not always stable. Under certain conditions the system continuously goes through cycles of increased and decreased cohesion (Figure 5a–c) in which the egalitarian state is gradually approached as one alliance eventually excludes all others. But once the egalitarian state is established (in Figure 5d, around time 5200), it quickly disintegrates because of internal conflicts between members of the winning alliance. Figure 5d illustrates one such cycle, showing that the dominant alliance remains relatively stable as long as the group excludes at least one member (“outsider”).

### Analytical approaches

Simple “mean-field” approximations help to understand model dynamics. These approximations focus on the average *a* and variance *v* of affinities computed over particular coalitions (see Text S1). For example, at an egalitarian state when all individuals have very high affinity to each other, the dynamics of *a* and *v* are predicted to evolve to particular stochastic equilibrium values, *a* ^*^ and *v* ^*^. The egalitarian state is stable if the fluctuations of pairwise affinities around *a* ^*^ do not result in negative affinities. We conjecture that the egalitarian state is stable if 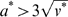, which is roughly equivalent to (*a* ^*^)^2^>10*v* ^*^, which in turn can be rewritten as
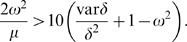
Here the mean *δ* and variance var*δ* are computed over the four *δ*-coefficients. Both the approximations and numerical simulations suggest that the egalitarian state cannot be stable with negative *δ*. Increasing the population size *N*, awareness *ω*, average *δ*, and decreasing the affinity decay rate *μ* and variance var*δ* all promote stability of the egalitarian state. The agreement of numerical simulations with analytical approximations is very good given the stochastic nature of the process. Similar approximations can be developed for other regimes. In particular, one can show (see Text S1) that the stabilizing effect of “outsiders” on the persistence of alliances is especially strong in small groups. This happens because successful conflicts against outsiders simultaneously increase the average *a* and decrease the variance *v* of the within-alliance affinities, with both effects being proportional to 1/*N*.

## Discussion

The overall goal of this paper was to develop a flexible theoretical framework for describing the emergence of alliances of individuals able to overcome the power of alpha-types in a population and to study the dynamics and consequences of these processes. We considered a group of individuals competing for rank and/or some limiting resource (e.g., mates). We assumed that individuals varied strongly in their fighting abilities. If all conflicts were exclusively dyadic and no coalitionary support was provided, a hierarchy would emerge with a few strongest individuals getting most of the resource [67]–[70]. However there is also a tendency (very small initially) for individuals to interfere in an ongoing dyadic conflict thus biasing its outcome one way or another. Positive outcomes of such interferences increase the affinities between individuals while negative outcomes decrease them. Using a minimum set of assumptions about cognitive abilities of individuals, we looked for conditions under which long-lasting coalitions (i.e. alliances) emerge in the group. We showed that such an outcome is promoted by increasing the frequency of interactions (which can be achieved in a number of ways) and decreasing the affinity decay rate. Most interestingly, the model shows that the shift from a state with no alliances to one or more alliances typically occurs in a phase-transition like fashion. Even more surprisingly, under certain conditions (that include some cultural inheritance of social networks) a single alliance comprising all members of the group can emerge in which the resource is divided evenly. That is, the competition among nonequal individuals can paradoxically result in their eventual equality. We emphasize that in our model, egalitarianism emerges from political dynamics of intense competition between individuals for higher social and reproductive success rather than by environmental constraints, social structure, or cultural processes. In other words, within-group conflicts promote the buildup of a group-level alliance. In a sense, once alliances start to form, there is no other reasonable strategy but to join one, and once social networks become highly heritable, a single alliance including all group members is destined to emerge.

Few clarifications are in order. First, in our model coalitionary interactions are mutually beneficial to all members of a coalition rather than altruistic (see ref.71 for a discussion of relevant terminology). We note that outside of humans there are not many examples of altruistic behavior towards genetically unrelated individuals without some direct fitness benefits present [72] with some of those that were initially suggested to be altruistic under closer examination turning out to be kin-directed or mutualistic [45], [73]. Even in humans certain behaviors that are viewed as altruistic may have a rather different origin. For example, food sharing may have originated as a way to avoid harassment, e.g. in the form of begging [45]. In any case, modern human behavior is strongly shaped by evolved culture [31] and might not be a good indicator of factors acting during its origin. Second, in our model we avoided the crucial step of the dominant game-theoretic paradigm which is an explicit evaluation of costs and benefits of certain actions in controlling one's decisions. In our model, coalitions and alliances emerge from simple processes based on individuals using only limited “local” information (i.e., information on own affinities but not on other individuals' affinities) rather than as a solution to an optimization task. Our approach is justified not only by its mathematical simplicity but by biological realism as well. Indeed, solving the cost-benefit optimization tasks (which require rather sophisticated algebra in modern game-theoretic models) would be very difficult for apes and early humans [45] especially given the multiplicity of behavioral choices and the dynamic nature of coalitions. Therefore treating coalitions and alliances in early human groups as an emergent property rather than an optimization task solution appears to be a much more realistic approach. We note that costs and benefits can be incorporated in our approach in a straightforward manner. Third, one should be careful in applying our model to contemporary humans (whether members of modern societies or hunter-gathers). In contemporary humans, an individual's decision on joining coalitions will be strongly affected by his/her estimates of costs, benefits, and risks associated as well as by cultural beliefs and traditions. These are the factors explicitly left outside of our framework.

Our results have implications for a number of questions related to human social evolution. The great apes' societies are very hierarchical; their social system is based on sharp status rivalry and depends on specific dispositions for dominance and submission. A major function of coalitions in apes is to maintain or change the dominance structure [1], [3]; although leveling coalitions are sometimes observed (e.g., [2]), they are typically of small size and short-lived. In sharp contrast, most known hunter-gatherer societies are egalitarian [8]–[10]. Their weak leaders merely assist a consensus-seeking process when the group needs to make decisions; at the band level, all main political actors behave as equal. It has been argued that in egalitarian societies the pyramid of power is turned upside down with potential subordinates being able to express dominance because they find collective security in a large, group-wide political coalition [10]. One factor that may have promoted transition to an egalitarian society is the development of larger brains and better political/social intelligence in response to intense within-group competition for increased social and reproductive success [16], [17], [25], [27]. Our model supports these arguments. Indeed, increased cognitive abilities would allow humans to maintain larger group sizes, have higher awareness of ongoing conflicts, better abilities in attracting allies and building complex coalitions, and better memories of past events. The changes in each of these characteristics may have shifted the group across the phase boundary to the regime where the emergence of an egalitarian state becomes unavoidable. [This discussion implies that the values of parameters characterizing cognitive abilities of apes and humans are located on different sides of the critical values identified above. Whether this assumption is justified is an important empirical question.] Similar effect would follow a change in mating system that would increase father-son social bonds, or an increase in fidelity of cultural inheritance of social networks. The fact that mother-daughter social bonds are often very strong suggests (everything else being the same) that females could more easily achieve egalitarian societies. The establishment of a stable group-wide egalitarian alliance should create conditions promoting the origin of conscience, moralistic aggression, altruism, and other cultural norms favoring the group interests over those of individuals [28]. Increasing within-group cohesion will also promote the group efficiency in between-group conflicts [29] and intensify cultural group selection.

In humans, a secondary transition from egalitarian societies to hierarchical states took place as the first civilizations were emerging. How can it be understood in terms of the model presented here? One can speculate that technological and cultural advances made the coalition size much less important in controlling the outcome of a conflict than the individuals' ability to directly control and use resources (e.g., weapons, information, food) that strongly influence conflict outcomes. In terms of our model, this would dramatically increase the variation in individual fighting abilities and simultaneously render the Lanchester-Osipov square law inapplicable, making egalitarianism unstable.

Besides developing a novel and general approach for modeling coalitionary interactions and providing theoretical support to some controversial verbal arguments concerning social transitions during the origin of humans, the research presented here allows one to make a number of testable predictions. In particular, our model has identified a number of factors (such as group size, the extent to which group members are aware of within-group conflicts, cognitive abilities, aggressiveness, persuasiveness, existence of outsiders, and the strength of parent-offspring social bonds) which are predicted to increase the likelihood and size of alliances and affect in specific ways individual social success and the degree of within-group inequality. Existing data on coalitions in mammals (in particular, in dolphins and primates) and in human hunter-gatherer societies should be useful in testing these predictions and in refining our model.

## Supporting Information

Text S1 (0.36 MB DOC)

Figure S1 (0.05 MB EPS)

Figure S2 (0.05 MB EPS)

Figure S3 (0.05 MB EPS)

Figure S4 (0.04 MB EPS)

Figure S5 (0.04 MB EPS)

Figure S6 (0.05 MB EPS)

Figure S7 (0.05 MB EPS)

Figure S8 (0.05 MB EPS)

Figure S9 (0.97 MB EPS)

Figure S10 (0.97 MB EPS)

## References

[1] Harcourt AH, de Waal FBM (1992). Coalitions and alliances in humans and other animals.

[2] Goodall J (1986). The chimpanzees of Gombe: patterns of behavior.

[3] de Waal FBM (2000). Chimpanzee Politics: Power and Sex among Apes.

[4] Widdig A, Streich WJ, Tembrock G (2000). Coalition formation among male Barbary macaques (*Macaca sylvanus*).. American Journal of Primatology.

[5] Vervaecke H, de Vries H, van Elsacker L (2000). Function and distribution of coalitions in captive bonobos (*Pan paniscus*).. Primates.

[6] Mitani JC, Amsler SJ (2003). Social and spatial aspect of male subgrouping in a community of wild chimpanzees.. Behaviour.

[7] Newton-Fisher NE (2004). Hierarchy and social status in Budongo chimpanzees.. Primates.

[8] Johnson AW, Earle T (1987). The evolution of human societies. From foraging group to agrarian state.

[9] Knauft BB (1991). Violence and sociality in human evolution.. Current Anthropology.

[10] Boehm C (1999). Hierarchy in the forest. The evolution of egalitarian behavior.

[11] Carneiro R (1970). A theory of the origin of the state.. Science.

[12] Rubin PH (2002). Darwinian politics: the evolutionary origin of freedom.

[13] Turchin P (2003). Historical Dynamics: Why States Rise and Fall.

[14] Turchin P (2005). War and Peace and War: The Life Cycles of Imperial Nations..

[15] Wright HT (1977). Recent research on the origin of the state.. Annual Review of Anthropology.

[16] Alexander RD (1990). How did humans evolve? Reflections on the uniquely unique species..

[17] Flinn MV, Geary DC, Ward CV (2005). Ecological dominance, social competition, and coalitionary arms races: why humans evolved extraordinary intelligence?. Evolution and Human Behavior.

[18] Jolly A (1966). Lemur social behavior and primate intelligence.. Science.

[19] Humphrey NK, Bateson PPG, Hinde RA (1976). The social function of intellect.. Growing Points in Ethology.

[20] Byrne RW, Whiten A (1988). Machiavellian intelligence. Social expertise and the evolution of intellect in monkeys, apes, and humans.

[21] Whiten A, Byrne RW (1997). Machiavellian intelligence II. Extensions and evaluations.

[22] Dunbar RIM (1998). The social brain hypothesis.. Evolutionary Anthropology.

[23] Dunbar RIM (2003). The social brain: mind, language, and society in evolutionary perspective.. Annual Review of Anthropology.

[24] Striedter GF (2005). Principles of brain evolution.

[25] Geary DC (2005). The origin of mind. Evolution of brain, cognition, and general intelligence.

[26] Roth G, Dicke U (2005). Evolution of the brain and intelligence.. Trends in Cognitive Sciences.

[27] Gavrilets S, Vose A (2006). The dynamics of Machiavellian intelligence.. Proceedings of the National Academy of Sciences USA.

[28] Boehm C (2007). Conscience origins, sanctioning selection, and the evolution of altruism in *Homo Sapiens*.. Current Anthropology.

[29] Wrangham RW (1999). Evolution of coalitionary killing.. Yearbook of Physical Anthropology.

[30] Choi JK, Bowles S (2007). The coevolution of parochial altruism and war.. Science.

[31] Richerson PJ, Boyd R (2005). Not by genes alone. How culture transformed human evolution.

[32] Kahan JP, Rapoport A (1984). The theory of coalition formation.

[33] Myerson RB (1991). Game theory. Analysis of conflict.

[34] Klusch M, Gerber A (2002). Dynamic coalition formation among rational agents.. IEEE Intelligent Systems.

[35] Konishi H, Ray D (2003). Coalition formation as a dynamic process.. Journal of Economic Theory.

[36] Noë R (1994). A model of coalition formation among male baboons with fighting ability as the crucial parameter.. Animal Behavior.

[37] Dugatkin LA (1998). A model of coalition formation in animals.. Proceedings of the Royal Society London B.

[38] Johnstone RA, Dugatkin LA (2000). Coalition formation in animals and the nature of winner and loser effects.. Proceedings of the Royal Society London B.

[39] Pandit SA, van Schaik CP (2003). A model of leveling coalitions among primate males: towards a theory of egalitarism.. Behavioral Ecology and Sociobiology.

[40] van Schaik CP, Pandit SA, Vodel ER (2004). A model for within-group coalitionary aggression among males.. Behavioral Ecology and Sociobiology.

[41] Whitehead H, Connor R (2005). Alliances I. How large should alliance be?. Animal Behavior.

[42] Connor R, Whitehead H (2005). Alliances II. Rates of encounter during resource utilization: a general model of intrasexual alliance formation in fission-fussion societies.. Animal Behavior.

[43] van Schaik CP, Pandit SA, Vodel ER, Kappeler PM, van Schaik CP (2006). Toward a general model for male-male coalitions in primate groups.. Cooperation in primates and humans.

[44] Mesterton-Gibbons M, Sherratt TN (2007). Coalition formation: a game-theoretic analysis.. Behavioral Ecology.

[45] Stevens JR, Cushman FA, Hauser MD (2005). Evolving the phychological mechanisms for cooperation.. Annual Review of Ecology and Systematics.

[46] Boyd R, Richerson PJ (1988). The evolution of reciprocity in sizable groups.. Journal of Theoretical Biology.

[47] Bach LA, Helvik T, Christiansen FB (2006). The evolution of n-player cooperation - threshold games and ESS bifurcations.. Journal of Theoretical Biology.

[48] Noë R, Harcourt AH, de Waal FBM (1992). Alliance formation among male baboons: shopping for profitable partners.. Coalitions and alliances in humans and other animals.

[49] Hammerstein P, Hammerstein P (2003). Why is reciprocity so rare in social animals? A protestant appeal.. Genetic and cultural evolution of cooperation.

[50] Skyrms B, Pemantle R (2000). A dynamic model of social network formation.. Proceedings of the National Academy of Sciences USA.

[51] Pemantle R, Skyrms B (2004). Network formation by reinforcement learning: The long and medium runs.. Mathematical Social Sciences.

[52] Pemantle R, Skyrms B (2004). Time to absorption in discounted reinforcement models.. Stochastic Processes and their Applications.

[53] Pacheco JM, Traulsen A, Nowak MA (2006). Active linking in evolutionary games.. Journal of Theoretical Biology.

[54] Santos FC, Pacheco JM, Lenaerts T (2006). Cooperation prevails when individuals adjust their social ties.. PLOS Computational Biology.

[55] Hruschka DJ, Henrich J (2006). Friendship, cliqueness, and the emergence of cooperation.. Journal of Theoretical Biology.

[56] Marcus J (1992). Political fluctuations in Mesoamerica.. National Geographic Research and Exploration.

[57] Iannone G (2002). Annales history and the ancient Maya state: some observations on the “dynamic model”.. American Anthropologist.

[58] Wittig RM, Crockford C, Seyfarth RM, Cheney DL (2007). Vocal alliances in Chacma baboons (*Papio hamadryas ursinus*).. Behavioral Ecology and Sociobiology.

[59] Kingman JFC (2002). Stochastic aspects of Lanchester's theory of warfare.. Journal of Applied Probability.

[60] Helmold RL (1993). Osipov: the ‘Russian Lanchester’.. European Journal of Operational Research.

[61] Wilson ML, Britton NF, Franks NR (2002). Chimpanzees and the mathematics of battle.. Proceedings of the Royal Society London B.

[62] Macy MW, Flache A (2002). Learning dynamics in social dilemmas.. Proceedings of the National Academy of Sciences USA.

[63] White KG (2001). Forgetting functions.. Animal Learning and Behavior.

[64] Newman MEJ (2003). The structure and function of complex networks.. SIAM Review.

[65] Connor RC, Heithaus MR, Barre LM (2001). Complex social structure, alliance stability and mating success in bottlenose dolphine ‘super-alliance’.. Proceedings of the Royal Society London B.

[66] Lusseau D, Wilson B, Hammond PS, Grellier K, Durban JW (2006). Quantifying the influence of sociality on population structure in bottlenose dolphines.. Journal of Animal Ecology.

[67] Landau HG (1951). On dominance relationships and the structure of animal societies.I. Effect on inherent charactreristics.. Bulletin of Mathematical Biophysics.

[68] Landau HG (1951). On dominance relationships and the structure of animal societies.I. Some effects on possible social factors.. Bulletin of Mathematical Biophysics.

[69] Bonabeau E, Theraulaz G, Deneubourg JL (1996). Mathematical model of self-organizing hierarchies in animal societies.. Bulletin of Mathematical Biology.

[70] Bonabeau E, Theraulaz G, Deneubourg JL (1999). Dominance orders in animal societies: the self-organization hypothesis revisited.. Bulletin of Mathematical Biology.

[71] West SA, Griffin AS, Gardner A (2007). Social semantics: altruism, cooperation, mutualism, strong reciprocity and group selection.. Journal of Evolutionary Biology.

[72] West SA, Griffin AS, Gardner A (2007). Evolutionary explanations for cooperation.. Current Biology.

[73] Kappeler PM, van Schaik CP (2006). Cooperation in primates and humans.

