# Supplementary material for: Dynamics of Alliance Formation and the Egalitarian Revolution
Source: PLoS One. 2008 Oct 1;3(10):e3293. doi: 10.1371/journal.pone.0003293 (PMC2547893; doi:10.1371/journal.pone.0003293)
Supplement: Text S1 — (0.36 MB DOC) [file pone.0003293.s001.doc]

## Supporting Information – Text S1

Here, we present

• some additional details on the computational methods used;

• a set of figures (Figures S1-S8) illustrating the effects of individual parameters on the coalitionary structure of the model;

• a set of figures (Figures S9 and S10) illustrating the effects of changes in multiple parameters simultaneously on the coalitionary structure of the model;

• • an outline of a mathematical method used to study the model analytically.

### Some details of computational methods

**Probabilities of help** For an individual *k* aware of a conflict between individuals *i* and *j*, the probabilities of helping to *i*, to *j*, and of no interference are set to and , respectively. In numerical simulations, we set


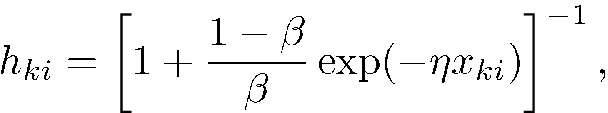


where $\beta$ and $\eta$ are scaling parameters. Note that $h_{\text{ki}}\to1$ for $x_{\text{ki}}\to\infty$, $h_{\text{ki}}\to0$ for $x_{\text{ki}}\to-\infty$, and $h_{\text{ki}}=\beta$ if $x_{\text{ki}}=0$.

**Numerical implementation** The model dynamics were simulated using Gillespie’s direct method (Gillespie 1977). That is, the next event to happen is chosen according to the corresponding rates. The time interval until the next event is drawn from an exponential distribution with a parameter equal to the sum of the rates of all possible events. All rates are recomputed after each event.

**Reference**

• Gillespie, D. T. Exact stochastic simulation of coupled chemical reactions. *Journal of Physical Chemistry* **81**, 2340-2361 (1977)

### Supplementary Figures and Legends

**Figures S1-S8.** To obtain Figures S1-S8 we performed 20 runs for each parameter combination. Each of the 20 runs was characterized by a single average value (computed over 100 observations taken from time 1000 to 2000). All plots correspond to the Tukey plots (i.e. show mean, min, max, lower, and upper quartiles). Other parameters were set to default values ().

**Figures S9 and S10.** To obtain Figures S9 and S10 we performed 40 runs for each parameter combination.

### Supplementary Methods: Mean field approximation for the dynamics of coalitions on the within-generation time-scale

We consider a group of *N* individuals in which conflicts occur at rate. Below we will use two types of averages: the average over a clique (i.e., a set of individuals who all are close allies), which we will denote as , and the average over all possible outcomes of the process, which we will denote as $\xi$ or $E\left( \xi\right)$, where $\xi$ is a random variable.

**Approximate dynamics of the mean and variance of affinities near an egalitarian state.** We assume that all *N* individuals are close allies so that each individual aware of a conflict interferes in it. The average affinity of the group is


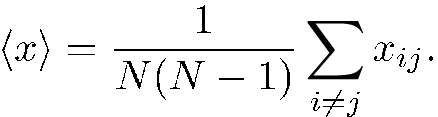


After each conflict, each affinity value changes from $x_{\text{ij}}$ to where $\varepsilon_{\text{ij}}$ is a random variable describing the change in affinity of individual *i* to individual *j*. Let be the expected average affinity. Since expectation and averaging are linear, the expected average affinity after a conflict can be written as


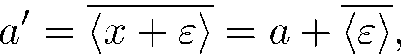


All affinities continuously decay to *0* at a constant rate $\mu$. Therefore, the dynamics of *a* are described by a differential equation


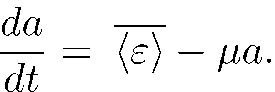
 (1)

Similarly, let be the expectation of the variance taken over all possible outcomes of the process. Then the variance after a conflict is


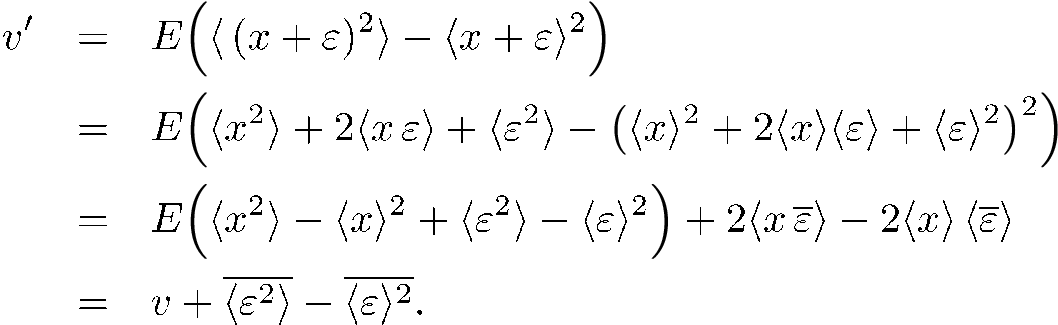


where, as an approximation, we assumed that $x$ and $\varepsilon$ are independent with respect to the averaging operator, i.e., .

All squares of affinities decay to *0* at a constant rate $2\mu$. Therefore, the dynamics of *v* are described by a differential equation


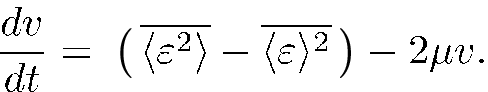
 (2)

First, we consider the expected change
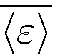
 in the affinity of a random pair of individuals after a conflict. There are three possibilities:

• With probability , the two individuals are the initiators of the conflict. Since either of the two initiators can be on the winning side, the expected change in their affinity is


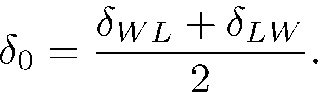


Under our assumptions about the meaning of parameters, $\delta_{0}$ is negative.

• With probability , one of the two individuals is an “initiator” while the other was aware of the conflict and interfered on behalf of one side. Since there are four ways to distribute the two individuals over the winning and losing coalitions and each occurs with equal probability, the expected change in their affinity is


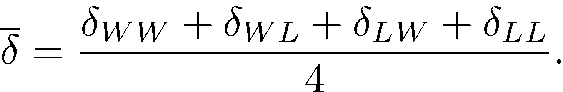


• With probability , neither individual is the initiator of the conflict but both are aware of it and interfere in the conflict. The expected change in their affinity is $\delta$.

Therefore,

(3)

Then, equations (S1,S3) predict that the average affinity in the egalitarian state evolves to an equilibrium value


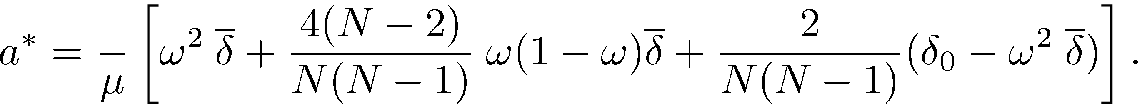
 (4)

The average affinity is positive only if $0\delta$. The last term in the brackets can be neglected relative to the first term even for small groups (e.g., *N* > 5). The second term in the brackets can be neglected for larger groups (e.g., *N* > 40) if $\omega$ is not too small. Under these conditions, .

In a similar way and using the results above,


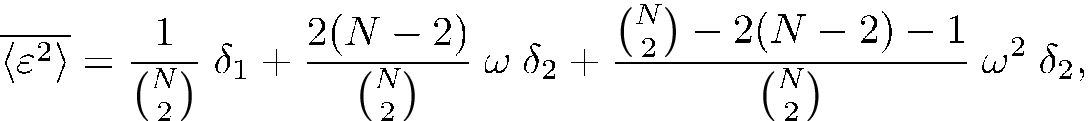
 (5)

where


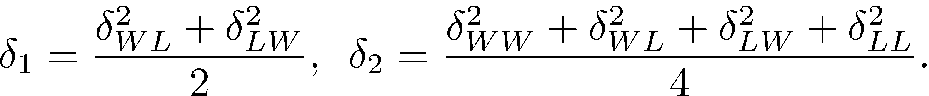


More involved calculations show that

where

The term $A_{1}$ can be interpreted as the expected value of for a random pair of individuals (*i* and *j*). There are three cases to consider.

• With probability , the focal individuals are the initiators of the conflict. In this case, $\text{Δ=}2\delta_{0}^{2}$.

• With probability , one of the two focal individuals is the initiator of the conflict while the other is aware of it.

• With probability , both focal individuals are aware of the conflict. In the last two cases, .

Therefore,


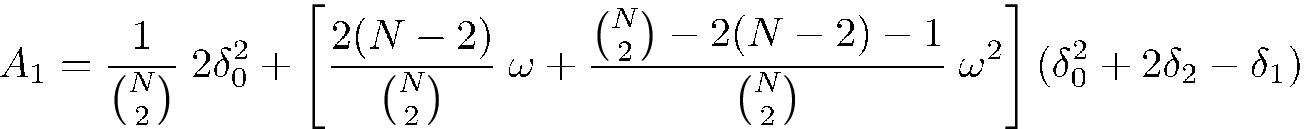
 (5)

The term $A_{2}$ can be interpreted as the expected value of for a random triple of individuals (*i*, *j* and *k*). There are three cases to consider.

• With probability , two of the three focal individuals are the initiators of the conflict while the third is aware of it. In this case,

• With probability , one of the three focal individuals is the initiator of the conflict while the two others are aware of it.

• With probability , none of the three focal individuals are the initiators of the conflict but all are aware of it.

To evaluate $\Delta$ in the last two cases, one needs to consider changes in affinities corresponding to all possible ways to assign three individuals to the winning and losing coalitions. This is done in the table below:

| winners | losers | 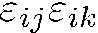 | 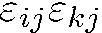 | 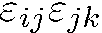 | 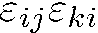 |
| --- | --- | --- | --- | --- | --- |
| 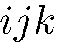 | - | 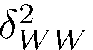 | 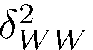 | 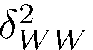 | 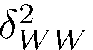 |
| 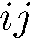 | 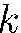 | 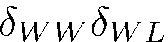 | 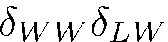 | 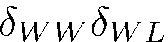 | 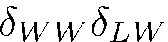 |
| 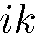 | 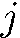 | 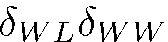 | 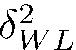 | 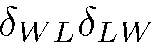 | 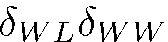 |
| 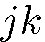 | 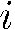 | 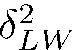 | 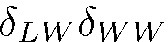 | 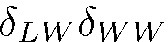 | 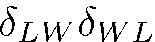 |
| 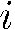 | 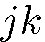 | 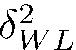 | 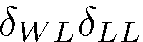 | 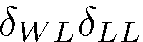 | 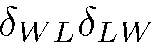 |
| 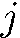 | 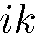 | 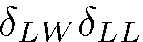 | 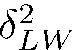 | 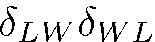 | 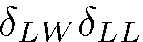 |
| 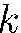 | 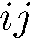 | 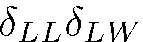 | 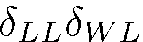 | 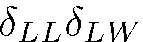 | 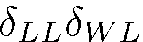 |
| 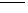 | 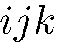 | 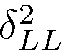 | 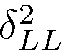 | 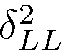 | 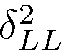 |

Using this table,


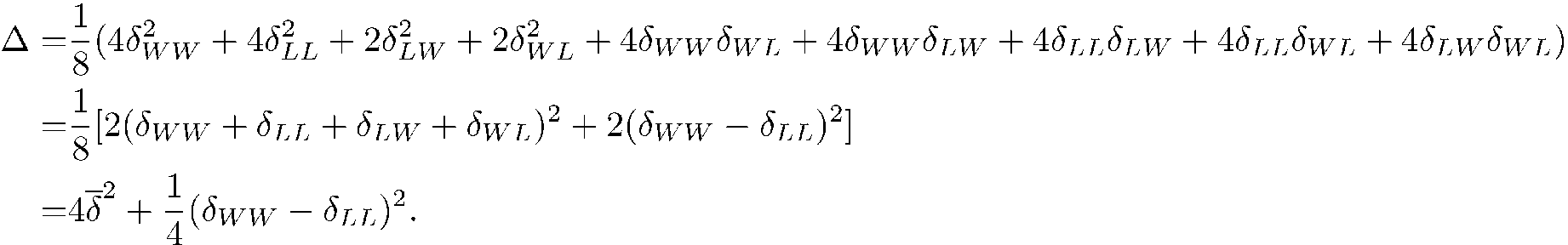


Therefore,


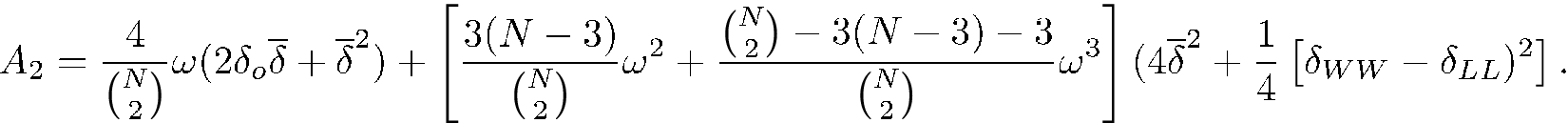
 (6)

The term $A_{3}$ can be interpreted as the expected value of for a random quartet of individuals (*i*, *j*, *k* and *l*). There are three cases to consider:

• With probability , two of the four focal individuals are the initiators of the conflict while the two others are aware of it. In this case,

• With probability , one of the four focal individuals is the initiator of the conflict while the three others are aware of it. In this case, .

• With probability , none of the three focal individuals are the initiators of the conflict but all are aware of it. In this case, .

Therefore,


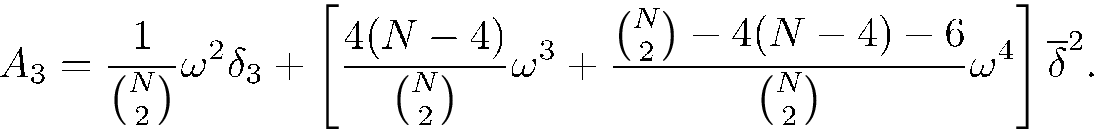
 (7)

Keeping only the leading terms in 1 / *N*, , which results in an equation for *v*:


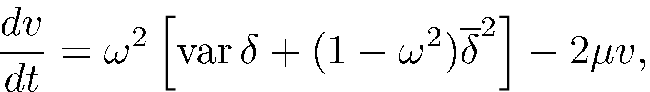
 (8)

where . Higher order corrections (in 1 / *N*) can be found in a straightforward way from the formula given above.

Keeping only the leading terms in 1 / *N*, the mean field approximation predicts the following equilibrium values at the egalitarian regime


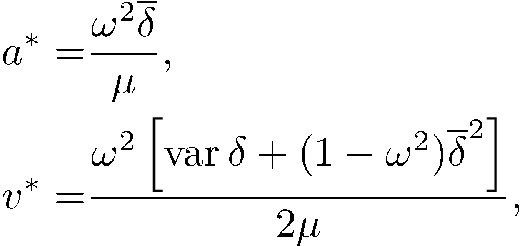


The egalitarian state is stable if the fluctuations of pairwise affinities around do not result in negative affinities. We conjecture that the egalitarian state is stable if , which is roughly equivalent to , which in turn can be rewritten as


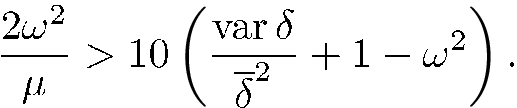


**The strongest clique comprising $N_{1}$ individuals; other $N_{2}\text{=N}-N_{1}$ individuals belong to weaker cliques.** We assume that all $N_{1}$ individuals in the clique are close allies that always help each other and never help outsiders. To evaluate the expected average over the clique , we need to find the expected value of $\text{Δ=ε}_{\text{ij}}$ for a random pair from the strongest clique. One needs to consider five possibilities:

• With probability , the focal individuals are the initiators of the conflict. In this case, $\text{Δ=δ}_{0}$.

• With probability , one of the focal individuals is an initiator of a conflict involving another member of the clique while the other is aware of the conflict and interferes on behalf of one side. In this case, .

• With probability , both focal individuals are aware of and interfere in a conflict between two other members of the clique. In this case, .

• With probability , one of the focal individuals is an initiator of a conflict involving an outsider while the other is aware of the conflict and interferes on behalf of the clique member. Assuming that the clique always wins, $\text{Δ=δ}_{\text{WW}}$.

• With probability , both focal individuals are aware of and interfere in a conflict between a member of the clique and an outsider. Assuming that the clique always wins, $\text{Δ=δ}_{\text{WW}}$.

Therefore,


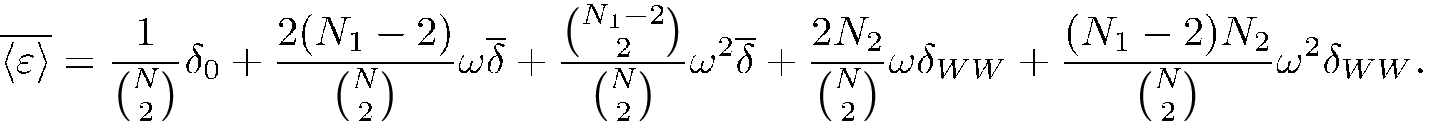
 (9)

Assume that (i.e., the single outsider case). Then the dynamics of the average within-clique affinity *a* are described by equation


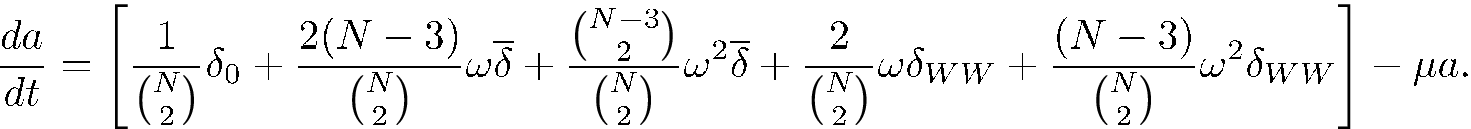


Thus, the average affinity under the single outsider regime is predicted to evolve to


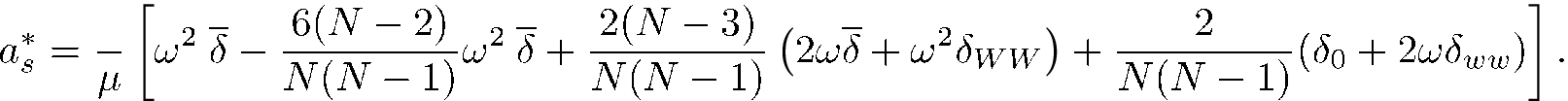


Keeping only terms of order *O*(1 / *N*) and larger in the brackets,


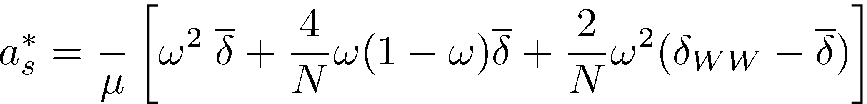
 (10)

It is illuminating to compare this expression with expression (S4) approximating the average affinity under egalitarian regime. Under the same assumptions, expression (S4) simplifies to


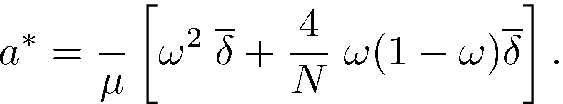
 (11)

If *N* is not too large, can be substantially smaller than . It is in this situation when a single outsider can have a strong stabilizing effect on a small coalition. For example, let and so that $=0.125\delta$. Then but , so that a single outsider significantly increases the average affinity of the clique. A single outsider will also reduce variance *v*, the effect of which will further strengthen the stability of the coalition.

**Supplementary Figure legends**

Figure S1. Effects of parameters , and ω on the proportion of individuals belonging to an alliance for a default set of parameter values.

Figure S2. Effects of parameters , and ω on the number of alliances for a default set of parameter values.

Figure S3. Effects of parameters , and ω on the size of the biggest alliance for a default set of parameter values.

Figure S4. Effects of parameters , and ω on the $C^{(1)}$ measure of the largest alliance for a default set of parameter values.

Figure S5. Effects of parameters , and ω on the $C^{(2)}$ measure of the largest alliance for a default set of parameter values.

Figure S6. Effects of parameters , and ω on the probability of help within of the largest alliance for a default set of parameter values.

Figure S7. Effects of parameters , and ω on the number of alliances with $C^{(1)}$>0.5 for a default set of parameter values.

Figure S8. Effects of parameters, and ω on the number of alliances with within-cluster probability of interference >0.5 for a default set of parameter values.

Figure S9. Effects of parameters *N*, β, μ, η, and ω on the number of individuals in alliances (first column) and the size of the largest alliance (second column) for First row: *N*=10, second row: *N*=20, third row: *N*=30. The values of the dependent variables are reflected both in the height of bars and in their color (as shown in the colorbars).

Figure S10. Effects of parameters N, β, μ, η, and ω on the measure $C^{(1)}$of the largest alliance (first column), and the number of alliances with $C^{(1)}$>0.5 (second column) for First row: N=10, second row: N=20, third row: N=30. The values of the dependent variables are reflected both in the height of bars and in their color (as shown in the colorbars).
